# Optimizing Flexible Microelectrode Designs for Enhanced Efficacy in Electrical Stimulation Therapy

**DOI:** 10.3390/mi15091104

**Published:** 2024-08-30

**Authors:** Lihong Qi, Zeru Tao, Mujie Liu, Kai Yao, Jiajie Song, Yuxuan Shang, Dan Su, Na Liu, Yongwei Jiang, Yuheng Wang

**Affiliations:** 1 Ningbo Zhenhai People’s Hospital Health Management Center, Ningbo 315202, China; 2 Department of Otorhinolaryngology Head and Neck Surgery, Ningbo Urology and Nephrology Hospital, Ningbo 315100, China; 3 Health Science Center, Faculty of Electrical Engineering and Computer Science, Ningbo University, Ningbo 315211, China; 4 Functional and Molecular Imaging Key Lab of Shaanxi Province, Department of Radiology, Tangdu Hospital, Air Force Medical University, Xi’an 710032, China; 5 Department of Nursing, Air Force Medical University, Xi’an 710032, China

**Keywords:** flexible, serpentine structure, MEMS, electrical stimulation therapy, chronic wound healing

## Abstract

To investigate the impact of electrode structure on Electrical Stimulation Therapy (EST) for chronic wound healing, this study designed three variants of flexible microelectrodes (FMs) with Ag-Cu coverings (ACCs), each exhibiting distinct geometrical configurations: hexagonal, cross-shaped, and serpentine. These were integrated with PPY/PDA/PANI (3/6) (full name: polypyrrole/polydopamine/polyaniline 3/6). Hydrogel dressing comprehensive animal studies, coupled with detailed electrical and mechanical modeling and simulations, were conducted to assess their performance. Results indicated that the serpentine-shaped FM outperformed its counterparts in terms of flexibility and safety, exhibiting minimal thermal effects and a reduced risk of burns. Notably, FMs with metal coverings under 3% demonstrated promising potential for optoelectronic self-powering capabilities. Additionally, simulation data highlighted the significant influence of hydrogel non-uniformity on the distribution of electrical properties across the skin surface, providing critical insights for optimizing EST protocols when employing hydrogel dressings.

## 1. Introduction

Chronic skin wounds, a prevalent medical concern, significantly deteriorate patient quality of life and impose substantial socioeconomic burdens, including increased healthcare costs, loss of productivity, and the financial strain on healthcare systems due to prolonged treatment and care requirements. The physiological response to skin injury involves the generation of an endogenous electric field (EEF), a critical factor in wound closure processes [1,2]. However, in chronic wounds, this natural EEF is often impaired or absent, necessitating alternative therapeutic interventions. Electrical Stimulation Therapy (EST) has emerged as a promising approach to facilitate wound healing. It aids in simulating the natural EEF, thus playing a pivotal role in mitigating bacterial proliferation, fostering cell migration, and stimulating the proliferation of fibroblasts. These processes are crucial in accelerating wound repair and compensating for the dysfunctional EEF inherent in chronic wounds [3,4,5,6].

In the realm of biomedical engineering, particularly in the development of devices for EST, flexible microelectrodes (FMs) have garnered considerable attention. Their diverse structures and functionalities make them highly suitable for such applications. Attributes such as reduced weight, smaller dimensions, and enhanced adaptability to the contours of the human body render these devices particularly advantageous [7,8]. The design of FMs is intricately linked to their flexibility and the distribution of stimulation, factors that can significantly impact the effectiveness of EST. Moreover, the safety of these devices is a paramount consideration, especially concerning the thermal effects associated with continuous current stimulation in wound therapy. In particular, DC stimulation poses a risk of thermal injury to the skin, underscoring the need for careful design and material selection in electrode construction [6,9].

Our research delves into the development and application of three distinct types of FMs, each featuring Ag-Cu coverings (ACCs) and adopting unique geometric configurations: hexagonal, cross-shaped, and serpentine. These designs were integrated with hydrogel technology, a novel approach in EST. This integration aimed to investigate the influence of electrode structure on wound healing efficiency and overall tissue regeneration. In addition, our study extends to the safety aspects of these electrode designs, a critical consideration in their clinical application. Employing a combination of experimental methods and computational simulations, we offer a comprehensive analysis of how different FM structures contribute to the efficacy and safety of EST in chronic wound management [4].

This introduction sets the stage for a detailed exploration of the intersection between advanced MEMS (Micro-Electro-Mechanical Systems) technology in electrode design and its application in medical therapy, particularly in the domain of wound healing. Our investigation aims to bridge the gap between engineering innovation and clinical application, offering insights that could transform the landscape of chronic wound management.

## 2. Materials and Methods

### 2.1. Fabrication of FMs

**Figure 1:**
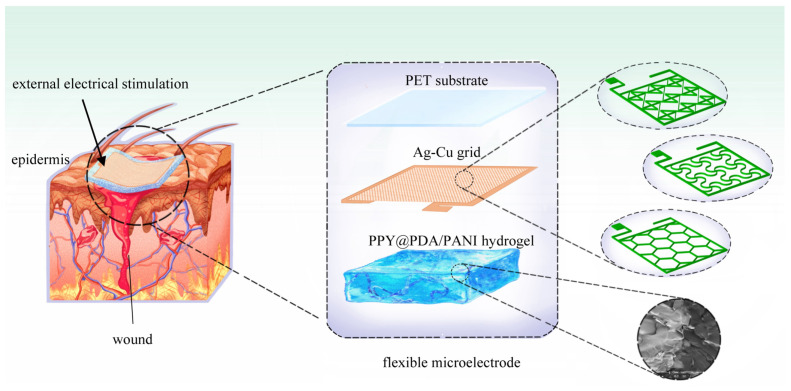
Schematic diagram of FMs’ structures of three types used for EST.

**Figure 2:**
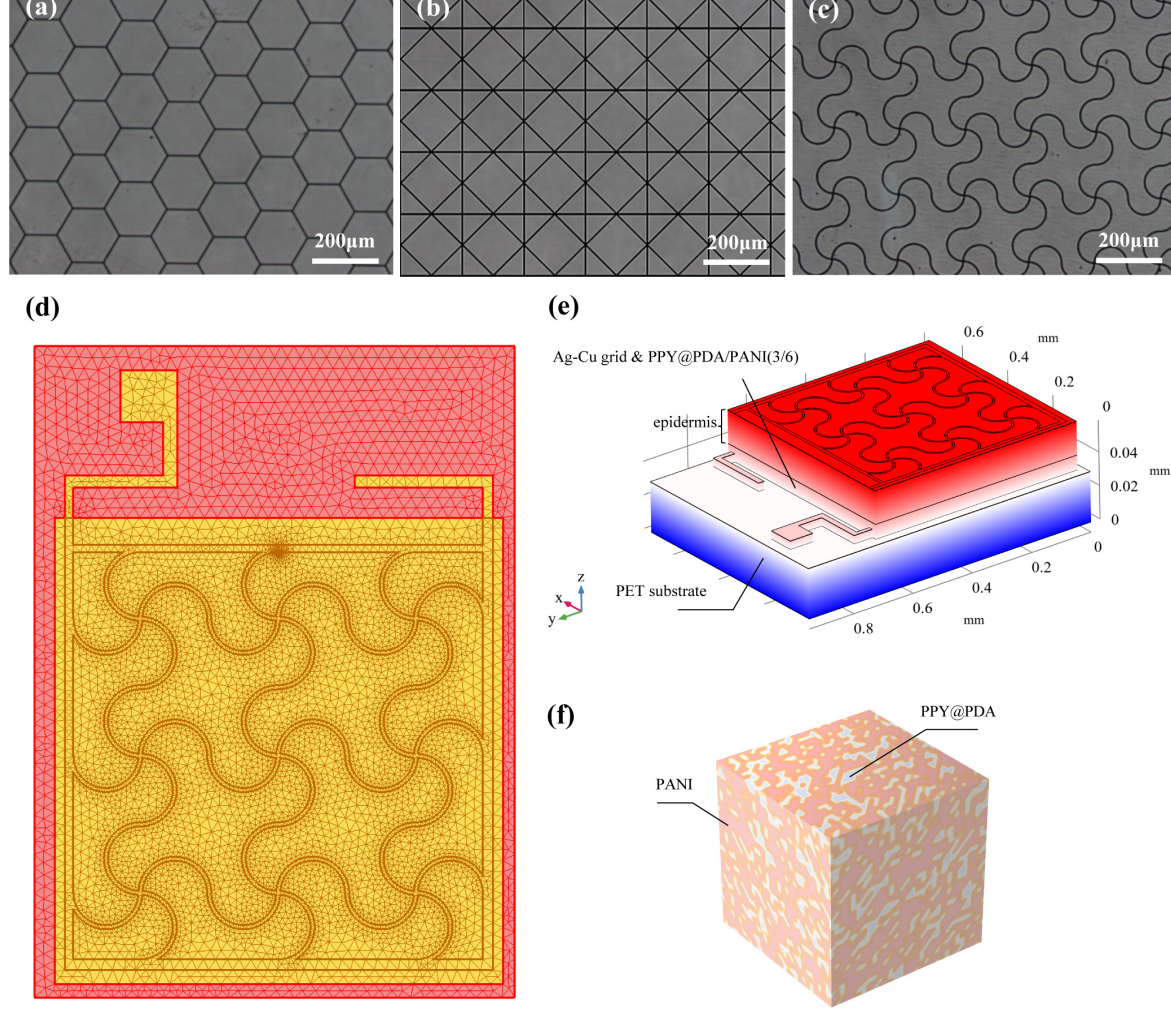
(**a**–**c**) Images of three FMs taken under optical microscope. (**d**) Top view of meshing before FEA. (**e**) Schematic diagram of simplified multi-layer model of FM–skin system. (**f**) Schematic diagram of PPY/PDA/PANI (3/6) (full name: polypyrrole/polydopamine/polyaniline 3/6) hydrogel.

The fabrication process of the flexible microelectrodes (FMs) commenced with the application of a UV photoresist onto a glass substrate using a doctor’s blade. This initial step was critical for ensuring a uniform coating. Subsequently, photolithography techniques were employed to intricately shape the UV photoresist film. This film served as a precise mask for the creation of a nickel master mold. The next phase involved patterning the poly (ethylene terephthalate) (PET) substrate. For this, the nickel master was accurately imprinted onto the UV glue previously coated on the PET substrate, which had a thickness of 120 μm. This process resulted in a mask-shaped pattern with a fine thickness of 3 μm on the substrate grid. Following this, silver nano-ink was meticulously filled into the formed grooves and then subjected to a sintering process at 150 °C for 15 min. This step was essential for ensuring the structural integrity and conductivity of the silver grid. The subsequent electroplating phase involved the application of a 2A current, facilitating the deposition of a dense copper layer over the silver grid. This copper covering, electroplated for approximately 5 min, was crucial to prevent any further oxidation of the electrodes. The final step in the fabrication process entailed the smoothing of the film’s surface. This was achieved by employing an aqueous solution containing fine silica particles, which helped in creating a more uniform and refined surface finish. Illustrations of this intricate process are provided for clarity: Figure 1 depicts the structured design of the FMs, while Figure 2a–c showcase the detailed images of the three types of flexible electrodes under an optical microscope, highlighting their distinct geometrical configurations [7].

### 2.2. Fabrication, Modeling, and Simulation of the FM–Hydrogel–Epidermis System (FHES)

For the modeling and analysis of the flexible microelectrodes (FMs) within the Flexible Microelectrode–Hydrogel–Epidermis System (FHES), a finite element analysis (FEA) approach was employed using COMSOL Multiphysics software (version 6.0). The detailed meshing of the serpentine FM, a crucial step in ensuring accurate simulation results, is depicted in Figure 2d. The hydrogel used in this study was primarily composed of polypyrrole/polydopamine (PPY/PDA) and polyaniline (PANI), but also included other components such as poly(vinyl alcohol) (PVA) for enhanced mechanical properties and biocompatibility. The crosslinking mechanism of the hydrogel involved the use of glutaraldehyde as a crosslinking agent, which facilitates the formation of a stable three-dimensional network structure [3,8].

The preparation of the hydrogel followed these steps:

1. Solution Preparation: A solution of poly(vinyl alcohol) (PVA) was prepared by dissolving PVA in deionized water with continuous stirring at 90 °C until a clear solution was obtained.
2. Polymer Addition: Polypyrrole/polydopamine (PPY/PDA) and polyaniline (PANI) powders were then gradually added to the PVA solution under vigorous stirring to ensure uniform dispersion.
3. Crosslinking: Glutaraldehyde was added to the mixture as a crosslinking agent. The solution was stirred continuously to ensure even crosslinking.
4. Curing: The mixture was poured into molds and allowed to cure at room temperature for 24 h, resulting in a stable hydrogel with desired mechanical and electrical properties.

The FEA methodology allowed for a thorough investigation of the complex interactions within the multi-layered structure of the FHES. The constructed model of the FHES, as shown in Figure 2e, consists of four distinct layers: the PET substrate, Ag-Cu coverings (ACCs), hydrogel, and epidermis. Each layer’s unique properties were taken into account to develop a comprehensive representation of the system. While the model parameters for the three electrode types were consistent, variations were introduced in the shapes of the ACCs to assess their impact on the system’s performance. Underpinning this model are fundamental principles from electrical, mechanical, and energy conversion theories. These principles were meticulously integrated to ensure a holistic and scientifically robust simulation environment, as detailed in Notes S1 and S2 in Supplementary Materials. A critical aspect of the model is the representation of the hydrogel layer. Hydrogels in this study were formulated as a non-uniformly doped mixture, incorporating components such as PPY/PDA and PANI. The doping and mixing of these components followed a specific ratio of 1:2, as shown in Figure 2f. This approach to hydrogel composition is pivotal as it leads to a dispersed distribution of internal conductivity, a factor crucial in simulating realistic interactions within the FHES. By incorporating these varied conductivity patterns, the model more accurately reflects the complex electrical behavior of the hydrogel layer, which is essential for understanding the overall efficacy of the FMs in EST applications.

### 2.3. Animal Experiment

**Figure 3:**
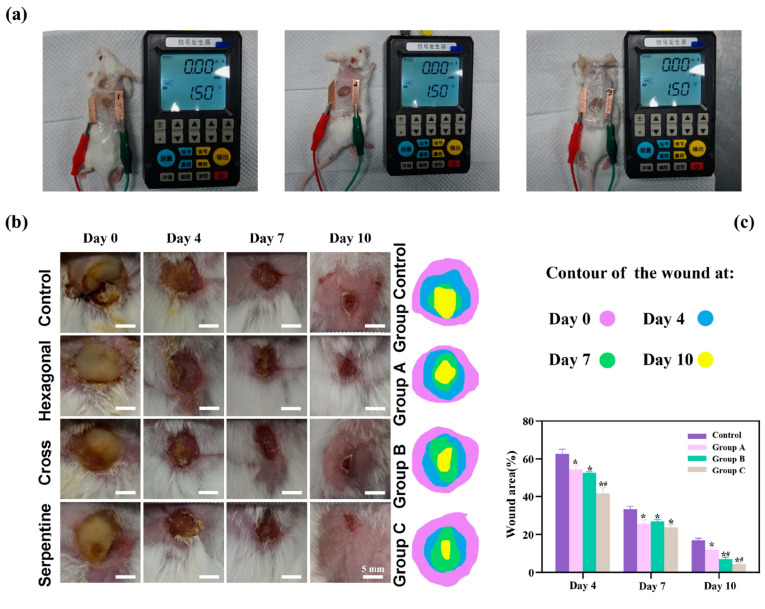
(**a**) Diagram of separate animal experiment. Here, we use different experimental animals to present stimulus. (**b**) Photographs of wound regions with different treatments. (**c**) Analysis of wound closure. Data are shown as mean ± SD (*n* = 3 per group; * *p* < 0.05 when compared with control group; # *p* < 0.05 when compared with Group A).

To rigorously assess the therapeutic efficacy of the three distinct types of flexible microelectrodes (FMs) with Ag-Cu coverings, a controlled animal study was meticulously designed, as detailed in Figure 3a. The species used for the in vivo experiments were Sprague-Dawley rats (male, over one year old), selected for their well-characterized wound healing properties and suitability for biomedical research.

Wound Infliction: The wounds were created using a biopsy punch under anesthesia to ensure uniformity and minimize pain. Each rat received a circular wound, 1 cm in diameter and 0.2 cm in depth, on its dorsal surface. Anesthesia was maintained throughout the procedure to ensure the welfare of the animals.

The experimental setup included a control group and three experimental groups, each corresponding to one of the three FM types (hexagonal, cross-shaped, and serpentine):

Control Group: Wounds treated using Tegaderm film dressings, a standard wound care approach, without the application of any electrical stimulation.

Group A: Wounds treated with hexagonal FMs integrated with hydrogel dressings.

Group B: Wounds treated with cross-shaped FMs integrated with hydrogel dressings.

Group C: Wounds treated with serpentine FMs integrated with hydrogel dressings.

Electrical Stimulation Therapy (EST) was applied to the experimental groups using the following parameters: a pulsed direct current (DC) with a frequency of 100 Hz and a voltage of 2 V was applied for 30 min daily.

The progression of wound healing was meticulously documented through serial photography at predetermined intervals (days 0, 3, 7, and 10). This visual record provided an empirical basis for evaluating the rate and extent of wound healing across the different groups. In addition to qualitative observations, a quantitative analysis was conducted to objectively measure and compare the healing outcomes, including the wound closure percentage and histological examination of the healed tissue. This dual approach, encompassing both visual and quantitative data, allowed for a comprehensive assessment of the therapeutic potential of each FM variant in the context of chronic wound management.

Management of Patch Dislodgement:

Throughout this study, the experimental patches were regularly monitored to ensure that they remained securely attached. If a patch became dislodged, it was immediately repositioned and re-secured using additional medical adhesive tape. Any dislodgement events were recorded, and the data from affected animals were carefully analyzed to determine if the dislodgement had any significant impact on the healing process.

## 3. Experimental Results

As shown by Figure 3b, there is a pronounced yellow purulent discharge in the control group in the early stages of wound healing, indicating a possible infection or inflammatory response. By contrast, the other three groups (hexagonal, cross-shaped, and serpentine FMs) exhibited relatively mild discharge, suggesting a reduced inflammatory response and better wound management. The histological analysis of wound tissue samples provided additional insights into the biological responses potentially influenced by different FM designs. It is suggested that wounds treated with the serpentine FM could have a higher density of fibroblasts and greater collagen deposition compared to those treated with hexagonal and cross-shaped FMs. These factors, known to be indicators of cell proliferation and migration, could align with the observed faster wound closure rates, which might indicate the potential superior efficacy of the serpentine design in promoting tissue regeneration and healing [6,10,11,12]. While direct experimental validation was not conducted in this study, these findings are consistent with the existing literature [6,10,11,12,13,14,15], which suggests that FMs with Ag-Cu coverings can enhance the healing rate of chronic wounds, and that the serpentine electrode may offer advantages in healing speed compared to the other two designs. These findings confirmed that FMs with Ag-Cu coverings better increase the healing rate of chronic wounds and the serpentine electrode showed a clear advantage compared with the other two types in terms of healing speed.

The results of the animal experiments demonstrated that all three types of flexible microelectrodes (FMs) with Ag-Cu coverings accelerated wound healing. On day 10, the wounds of the rats treated with the serpentine FM achieved a high closure ratio of 91%, while the wounds in the control group remained largely unhealed (less than 80%) (Figure 3c). In addition, the wounds treated with the hexagonal and cross-shaped electrode achieved the closure ratios of 85% and 90%, respectively [10,11,16]. To clearly compare the physical properties’ distribution of the epidermis, a 0.6 mm × 0.65 mm part of the cell structure was selected in the simulation, and the simulation results were normalized [12].

**Figure 4:**
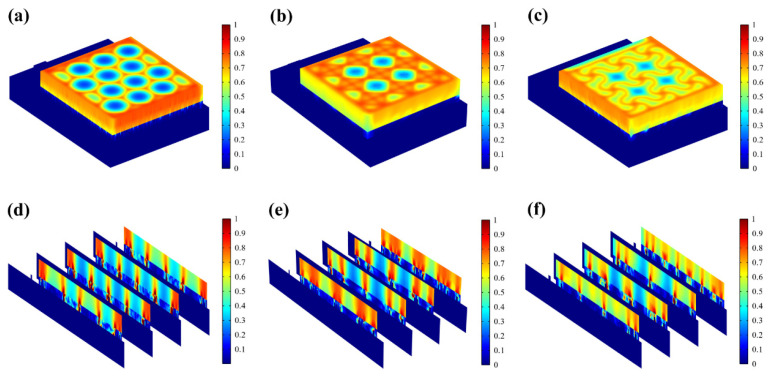
(**a**–**c**) Normalized resistive loss distribution of hexagonal, cross-shaped, and serpentine FHES. (**d**–**f**) Corresponding partial longitudinal section distribution of hexagonal, cross-shaped, and serpentine FHES.

**Figure 5:**
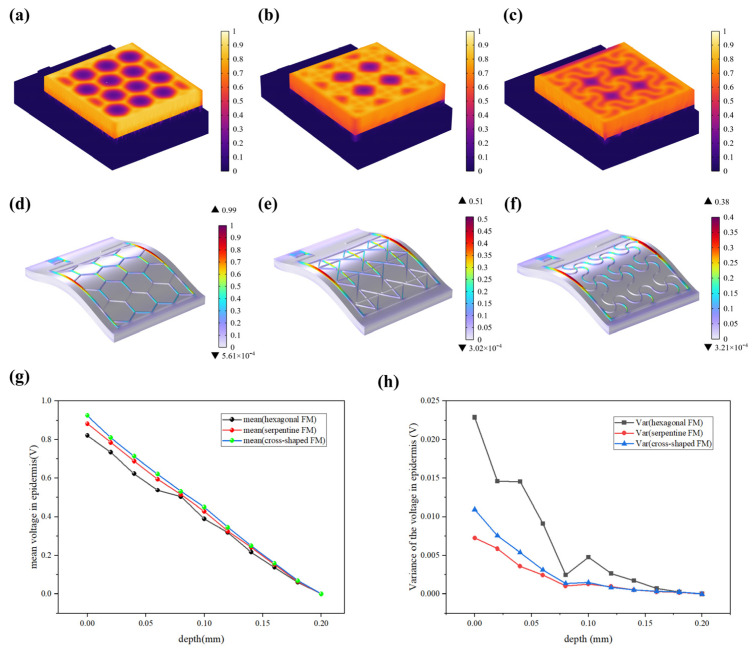
(**a**–**c**) Normalized thermal accumulation rate of three types of FHES. (**d**–**f**) Normalized force distribution of three types of FMs. (**g**) Normalized average voltage distribution of three FMs at different depths in epidermis. (**h**) Normalized voltage variance distribution.

The resistive loss distribution (Figure 4a–c) is highly correlated with the heat accumulation rate (Figure 5a–c). Normalized results showed that the epidermis accumulated heat faster when hexagonal and cross-shaped FMs were applied, especially at the cross point, indicating risks of burns (Figure 5a,b). While for serpentine FM, heat accumulation displayed a more balanced distribution (Figure 5c).

## 4. Experimental Discussion

Most of the energy transferred to the epidermis from electrical stimulation is converted to heat. As far as safety is concerned, the high impedance of the epidermis leads to thermal accumulation, which may cause risks of burns. Figure 5d–f revealed that the stress of the ACC is larger compared with that of PET. Therefore, the structure of ACC is the dominant factor affecting the flexibility of FMs. The maximum stress of serpentine FM (Figure 5f) is lower, which is 39% of hexagonal FM (Figure 5d) and 75% of cross-shaped FM (Figure 5e), denoting its optimal flexibility. Consequently, the serpentine FM fits more closely with the skin surface; thus, the air resistive loss caused by the loose fitting is less and the external electrical stimulation can be better transmitted to the skin surface.

The use of Ag-Cu coverings was chosen due to their excellent electrical conductivity, antibacterial properties, and biocompatibility. Silver (Ag) has long been known for its potent antibacterial effects, which can help prevent infections in wound care applications. Copper (Cu) also possesses antimicrobial properties and contributes to the overall conductivity of the electrode. The combination of Ag and Cu provides a balance of high electrical performance and infection control, making it ideal for use in EST.

The specific geometric configurations (hexagonal, cross-shaped, and serpentine) were selected to evaluate the impact of electrode design on the distribution of electrical stimulation and mechanical flexibility. The serpentine design, in particular, was hypothesized to offer superior flexibility and conformability to irregular skin surfaces, which is crucial for effective wound treatment. The hexagonal and cross-shaped designs were included to compare different structural approaches and to identify the most efficient configuration for EST.

Additionally, the air impedance is high; if the contact between FMs and the skin surface is not tight enough, the heat accumulation caused by high-impedance air will aggravate the possibility and severity of skin burns. Therefore, the high flexibility of the serpentine structure also contributes to the safety of EST [17,18,19]. The pronounced yellow purulent discharge observed in the control group indicates a possible infection or inflammatory response, which raises concerns about wound management and potential sepsis. However, the experimental groups treated with flexible microelectrodes (FMs) integrated with hydrogel dressings showed relatively mild discharge, suggesting a more controlled inflammatory response and effective infection prevention. This could be attributed to the antibacterial properties of the Ag-Cu coverings and the hydrogel’s ability to maintain a moist wound environment, which is conducive to healing and reduces the risk of infection. It is crucial to monitor for signs of infection and sepsis following electrode/hydrogel insertion and subsequent electrical stimulation. While this study did not observe significant adverse effects related to infection in the experimental groups, it is recommended that future studies include comprehensive microbial assessments and long-term monitoring to ensure the safety and biocompatibility of these devices. This multidisciplinary approach will help in understanding the full implications of using flexible microelectrodes in chronic wound management and ensuring patient safety.

**Figure 6:**
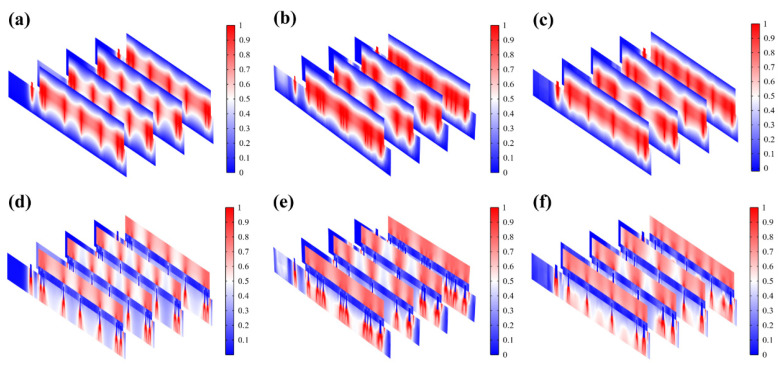
(**a**–**c**) Normalized electric potential distribution of hexagonal, cross-shaped, and serpentine FHES. (**d**–**f**) Normalized electric field distribution of hexagonal, cross-shaped, and serpentine FHES.

Figure 5g demonstrates that EST compensates well for the loss of endogenous electric potential when working with the highly conductive hydrogel. The electric potential distribution of serpentine FM is more uniform, with a normalized variance of 32% of the hexagonal one and 75% of the cross-shaped one (Figure 5h and Figure 6a–c). Also, the electric field (Figure 6d–f) maintains its intensity inside the skin, playing a key role in compensating for the endogenous electric field. This consistent and uniform electric field is crucial for enhancing cell migration and proliferation, especially when the endogenous electric field is disrupted. The serpentine FM’s design likely optimizes the therapeutic effects of EST by providing stable electrical conditions, which can accelerate wound healing and improve overall treatment efficiency.

**Figure 7:**
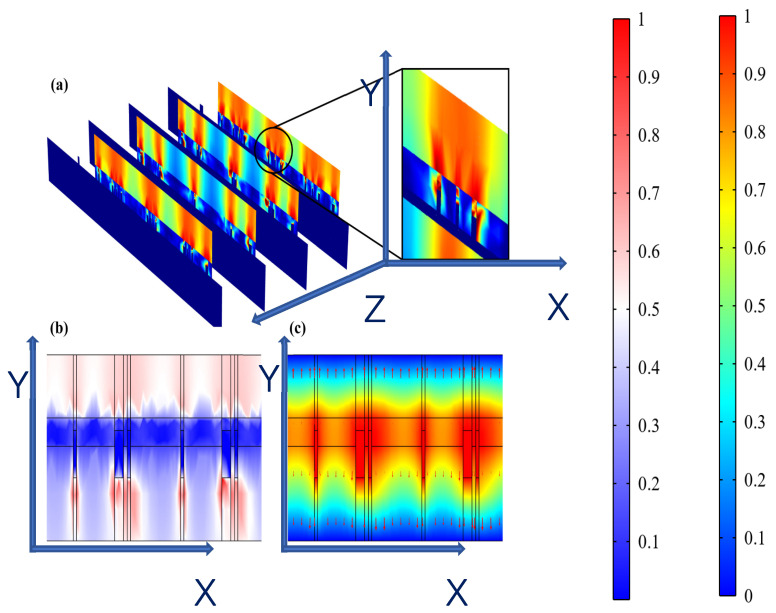
(**a**) Dispersed resistive loss distribution of FHES. (**b**) Dispersed electric field distribution of FHES. (**c**) Electrical potential distribution of FHES (X and Y represents section plane, and Z is perpendicular to section plane).

It was observed from Figure 7a,b that hydrogel showed a dispersed resistive loss distribution and electric field distribution due to the random doping, which also had an impact on the distribution in the epidermis. However, the voltage distribution did not show an obviously dispersed distribution in the hydrogel and epidermis (Figure 7c) because the voltage is an integral of the potential and the discrete type is greatly reduced. Therefore, the voltage is minimally affected by non-uniform doping, which may offer guidance for EST when working with hydrogel dressings where the composition and structure of hydrogels affect the epidermal electrical stimulation. Less than 3% of the substrate is covered with the ACCs, causing low optical losses, which indicates that the designed FMs have great potential for optoelectronic self-power [1,20].

## 5. Conclusions

In conclusion, this study decisively establishes that the geometry of flexible microelectrodes (FMs) is crucial in optimizing the efficacy of Electrical Stimulation Therapy (EST) for chronic wound healing. Our comparative analysis of hexagonal, cross-shaped, and serpentine FMs indicates that the serpentine design is superior, demonstrating enhanced wound healing efficiency. Specifically, the serpentine FM exhibited a high wound closure ratio of 91% by day 10, compared to 85% for the hexagonal FM and 90% for the cross-shaped FM. The histological analysis further confirmed that the serpentine FM facilitated a more effective promotion of chronic wound healing, as we previously reported in the literature, evidenced by increased fibroblast activity and collagen deposition observed in the wound tissue. These results underscore the potential of the serpentine design in promoting faster and more efficient wound healing.

This is attributed to its ability to more uniformly distribute electrical stimulation across the wound surface, thereby compensating for the deficient endogenous electric field in chronic wounds. Notably, the serpentine FM also exhibits a lower thermal accumulation rate, significantly reducing burn risks, a critical safety consideration in EST. Furthermore, its mechanical properties, with a markedly lower stress level compared to the other designs, ensure better adaptability to skin contours, enhancing treatment effectiveness and patient comfort. Additionally, this study highlights the impact of hydrogel non-uniformity on the electrical properties of skin, suggesting the need for the careful consideration of hydrogel composition in EST applications. The minimal coverage of the substrate by Ag-Cu coverings (less than 3%) in the serpentine FM design also hints at its potential for optoelectronic self-powering capabilities, opening new avenues for self-sustaining therapeutic devices. Collectively, these findings underscore the significance of FM design in advancing the field of bioelectronic medicine, particularly in the context of chronic wound management.

## Data Availability

Access to all data may be made on reasonable request to the corresponding authors.

## Supplementary Materials

The following supporting information can be downloaded at: https://www.mdpi.com/article/10.3390/mi15091104/s1, Note S1. Electrical modelling of simplified multi-layer flexible microelectrodes -hydrogel-epidermis system (FHES); Note S2. Structural mechanics modelling of FMs; Figure S1. Images of PPY@PDA/PANI (3/6) hydrogel under an electron microscope; Figure S2. (a–c) Normalized electric potential distribution of the hexagonal, cross-shaped and serpentine FHES. (d–f) Normalized electric field distribution of the hexagonal, cross-shaped and serpentine FHES.
