# Supplementary material for: Optimizing Flexible Microelectrode Designs for Enhanced Efficacy in Electrical Stimulation Therapy
Source: Micromachines (Basel). 2024 Aug 30;15(9):1104. doi: 10.3390/mi15091104 (PMC11434305; doi:10.3390/mi15091104)
Supplement: Supplementary file 1 [file micromachines-15-01104-s001.zip › micromachines-3066685-supplementary.pdf]

micromachines

Supplementary information

Optimizing Flexible Microelectrode Designs for Enhanced Efficacy in Electrical Stimulation Therapy

Lihong Qi [1,†], Zeru Tao [2,†], Mujie Liu [3], Kai Yao [3], Jiajie Song [4], Yuxuan Shang [4], Dan Su [4], Na Liu [4,5], Yongwei Jiang [1,*] and Yuheng Wang [4,*]

[1] Ningbo Zhenhai People's Hospital Health Management Center, Ningbo 315202, China
[2] Department of Otorhinolaryngology Head and Neck Surgery, Ningbo Urology and Nephrology Hospital, Ningbo 315100, China
[3] Health Science Center, Faculty of Electrical Engineering and Computer Science, Ningbo University, Ningbo 315211, China
[4] Functional and Molecular Imaging Key Lab of Shaanxi Province, Department of Radiology, Tangdu Hospital, Air Force Medical University, Xi'an 710032, China
[5] Department of Nursing, Air Force Medical University, Xi'an 710032, China
* Correspondence: yongwei086@163.com (Y.J.); nano.plasma.uv@gmail.com (Y.W.)
† These authors contributed equally to this work.

Note S1. Electrical modelling of simplified multi-layer flexible microelectrodes-hydrogel-epidermis system (FHES)

The charge migrates from the Ag-Cu coverings (ACCs) to hydrogel and then to the skin surface (epidermis). According to Ampere circuit rule and Gauss's law in Maxwell's equations, the current continuity equation can be deduced as follows:

$$\vec{\nabla} \cdot \vec{J} = -\frac{\partial \rho_V}{\partial t} \tag{1}$$

where $\vec{J}$ is the current density in the medium and ρ_V is the charge density.

In the domain with external charge input, Poisson equation is introduced to describe the relationship between potential distribution and external charge density. For example, external current and charge transportation exist in the metal wire lead-out.

$$\vec{\nabla}^2 \varphi = -\frac{\rho_V}{\varepsilon_0 \varepsilon_r} \tag{2}$$

where φ is electric potential, ε_0 the permittivity of the vacuum, ε_r the relative permittivity of Ag (Because the dense copper layer is electroplated on the surface of the silver grid).

In other domains without external charge input, Poisson's equation can be simplified as Laplace's equation.

$$\vec{\nabla}^2 \varphi = 0 \tag{3}$$

Constitutive relationship between current density and electric field can be described as

$$\vec{J} = \sigma \vec{E} \tag{4}$$

where σ is the conductivity of medium. Conductivity differs between skin, hydrogel and PET. The internal conductivity of hydrogel also shows differences due to the non-uniform doping components inside.

Electric field $\vec{E}$ is defined in terms of the applied voltage φ

$$\vec{E} = -\vec{\nabla} \cdot \varphi \tag{5}$$

As for the boundary conditions, a constant voltage of 1 V is applied to the output of the ACCs' lead. The lower bottom surface of the PET substrate is in contact with air, and the air conductivity tends to 0, so the interface is approximated as an insulating surface. The distal end of the skin is approximated as having no charge movement and a potential of 0, which is approximated as grounded.

With the above equation (1) to (5) and boundaries, we obtain the distribution of electric potential, electric field as well as resistive loss using finite element analysis of COMSOL.

Note S2. Structural mechanics modelling of FMs

Newton's second law of motion can be expressed as

$$\vec{\nabla}\cdot\vec{\sigma}+\vec{f}=\rho\frac{\partial^2\vec{u}}{\partial t^2} \tag{6}$$

Considering the steady-state equilibrium conditions and neglect the intermediate process, the equation can be simplified as

$$\vec{\nabla}\cdot\vec{\sigma}+\vec{f}=0 \tag{7}$$

where $\vec{\sigma}$ is internal stress and $\vec{f}$ is external force per unit volume applied to the system.

Stress and strain can be expressed by 3x3 matrix

$$\sigma=\begin{bmatrix}\sigma_{xx} & \sigma_{xy} & \sigma_{xz}\\ \sigma_{yx} & \sigma_{yy} & \sigma_{yz}\\ \sigma_{zx} & \sigma_{zy} & \sigma_{zz}\end{bmatrix},\varepsilon=\begin{bmatrix}\varepsilon_{xx} & \varepsilon_{xy} & \varepsilon_{xz}\\ \varepsilon_{yx} & \varepsilon_{yy} & \varepsilon_{yz}\\ \varepsilon_{zx} & \varepsilon_{zy} & \varepsilon_{zz}\end{bmatrix}$$

Equation of strain compatibility is introduced to describe the relationship between strain and displacement.

$$\begin{bmatrix}\varepsilon_{xx}\\ \varepsilon_{yy}\\ \varepsilon_{zz}\\ \varepsilon_{xy}\\ \varepsilon_{yz}\\ \varepsilon_{xz}\end{bmatrix}=\begin{bmatrix}\partial u/\partial x\\ \partial v/\partial y\\ \partial w/\partial z\\ 1/2\left(\partial u/\partial y+\partial v/\partial x\right)\\ 1/2\left(\partial v/\partial z+\partial w/\partial y\right)\\ 1/2\left(\partial u/\partial z+\partial w/\partial x\right)\end{bmatrix} \tag{8}$$

We assume that both materials forming the FMs and skin are linear elastic materials. Consequently, according to Hooke's law and connection between elastic modulus we obtain that

$$\begin{bmatrix}\sigma_{xx}\\ \sigma_{yy}\\ \sigma_{zz}\\ \sigma_{xy}\\ \sigma_{yz}\\ \sigma_{xz}\end{bmatrix}=\frac{E}{(1+\nu)(1-2\nu)}=\begin{bmatrix}1-\nu & 0 & 0 & 0 & 0 & 0\\ 0 & 1-\nu & 0 & 0 & 0 & 0\\ 0 & 0 & 1-\nu & 0 & 0 & 0\\ 0 & 0 & 0 & (1-2\nu)/2 & 0 & 0\\ 0 & 0 & 0 & 0 & (1-2\nu)/2 & 0\\ 0 & 0 & 0 & 0 & 0 & (1-2\nu)/2\end{bmatrix}\begin{bmatrix}\sigma_{xx}\\ \sigma_{yy}\\ \sigma_{zz}\\ \sigma_{xy}\\ \sigma_{yz}\\ \sigma_{xz}\end{bmatrix} \tag{9}$$

Also, the initial condition of displacement and external force that applied are given in equation(11). The initial displacement and velocity of the electrode is 0. The entire lower surface of the electrode is subjected to an external force of magnitude $100\,N/m^2$ along the z-axis upward. In addition, the long side of the electrode can be deformed and displaced, while the short side is fixed and the displacement is constant to zero.

$$\begin{cases}u_x=u_y=u_z=0\\ \dfrac{\partial u_x}{\partial t}=\dfrac{\partial u_y}{\partial t}=\dfrac{\partial u_z}{\partial t}=0\\ f_x=f_y=0\\ f_z=100\,N/m^2\end{cases} \tag{10}$$

With equations above and initial conditions given, we obtain the corresponding distribution of stress and displacement in steady state, which is effective to evaluate the flexibility of bioelectrode.

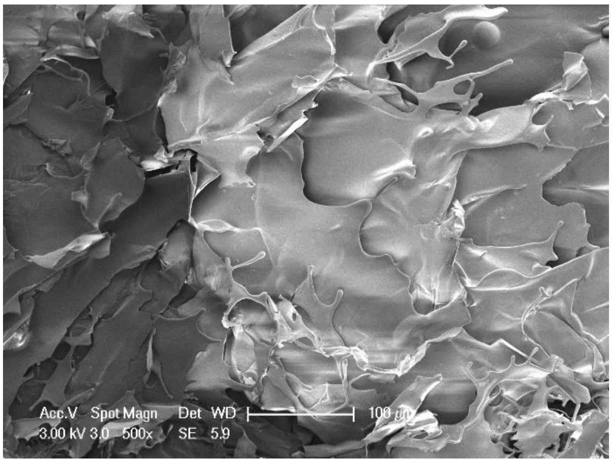

Figure S1. Images of PPY@PDA/PANI (3/6) hydrogel under an electron microscope.

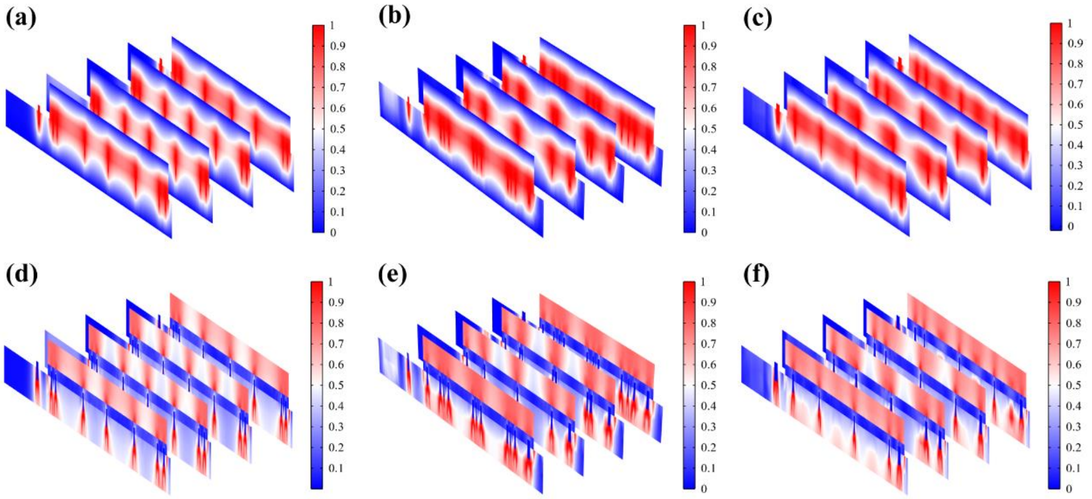

Figure S2. (a)~(c) Normalized electric potential distribution of the hexagonal, cross-shaped and serpentine FHES. (d)~(f) Normalized electric field distribution of the hexagonal, cross-shaped and serpentine FHES.
